# Mystery in experimental psychology, how to measure aesthetic emotions?

**DOI:** 10.3389/fpsyg.2014.01006

**Published:** 2014-09-10

**Authors:** Leonid Perlovsky

**Affiliations:** Athinoula A. Martinos Center for Biomedical Imaging, Harvard UniversityCharlestown, MA, USA

**Keywords:** aesthetic emotions, beautiful, cognitive dissonance, prosody, music, mind, mathematical models, experimental measures

Measuring aesthetic emotions presents difficulties and this article aims at challenging experimental community of psychologists and cognitive scientists to address this challenge. I discuss possible cognitive mechanisms of aesthetic emotions, existing results, difficulties, and opportunities.

## Aesthetic emotions

Emotions related to knowledge have been called aesthetic since the time of Kant. Existence of these specific emotions has been experimentally demonstrated and their cognitive functions have been analyzed (Perlovsky et al., 2010, 2013; Masataka and Perlovsky, 2012, 2013; Perlovsky, 2014). Musical emotions, an ability to be affected by sounds, the reason for evolutionary emergence of this ability were called “the greatest mystery” by Darwin (1871). Aesthetic emotions include musical emotions, emotions of cognitive dissonances, emotions related to improving knowledge, in particular improving knowledge near the “top” of the mental hierarchy experienced as emotions of the beautiful (Perlovsky, 2014). Other fundamentally important aesthetic emotions are discussed later.

Although a number of outstanding scientists work in this field, there are no methods that even come close to a universally agreed approach to measuring qualities of aesthetic emotions, or establishing their classification, or an approximate number. Some researchers accept that there are two fundamental emotional dimensions, arousal and valence, and the rest are mixtures of the two (Russell, 1980). Juslin (2013) suggests that there are no specifically musical emotions, emotions experienced while listening to music are the same as those described by standard emotional words and mixtures of them. Scherer (2005) maintains that there are specifically musical emotions, the number of emotions is very large, however he doubts that they could be measured and that such measurements could be useful. Many researchers (Zentner et al., 2008) suggest that there is a tremendous number of aesthetic emotions and develop approaches to their measurements (e.g., tenderness, transcendence, nostalgia). The author of this article (Perlovsky, 2014) suggests specific and fundamental cognitive functions of musical emotions, which qualities and numbers are beyond possible language descriptions.

The theory of drives and emotions (Grossberg and Levine, 1987) suggests that emotions and related feelings correspond to satisfaction or dissatisfaction of drives and instincts. These measure vital bodily parameters (such as sugar level in blood), and emotional neural signals convey their satisfactory or unsatisfactory ranges to decision-making parts of the brain. These are “bodily” emotions, of ancient origins, and there are words in every language for describing them. In English there are approximately 150 emotional words (Shaver et al., 1987); between 5 and 14 of these, are identified as significantly different by various researchers (Scherer, 2005; Petrov et al., 2012).

The Grossberg-Levine theory has been extended to aesthetic emotions (Perlovsky and McManus, 1991; Perlovsky, 2001, 2007, 2010, 2014). The knowledge instinct has been suggested to drive improvement of mental representations in their correspondence to objects and events in the world (knowledge). In addition to increasing knowledge, the knowledge instinct drives the brain-mind to resolve contradictions between knowledge and bodily instincts, and among various aspects of knowledge. Satisfaction or dissatisfaction of this instinct is experienced as aesthetic emotions. A combinatorially large number of potential contradictions in knowledge predicts a very large number of emotions of cognitive dissonance and musical emotions. Musical emotions have been predicted to help overcoming emotional contradictions of cognitive dissonances among elements of knowledge and accumulate contradictory knowledge (Perlovsky, 2010, 2012a,b, 2014). These predictions have been experimentally confirmed (Masataka and Perlovsky, 2012, 2013; Perlovsky et al., 2013; Perlovsky, 2014).

Prosodial emotions that we hear in human voice are usually discussed in their ancient and primitive aspects, which unify us with pre-language animals, such as signals of danger, rage, anger, disgust, and happiness. Less discussed aesthetic emotions of prosody are specifically human emotions motivating us to connect sounds and meanings in speech or more generally in language (although emotions of prosody are contained in sounds, we used to associate them with language). These emotions sound usually below the level of consciousness in everyday unarticulated speech. They constitute the essence of poetry beginning before the Bible, Homer, or Koran. Despite the importance of these emotions they have not been sufficiently studied. Not a single experimental publication addressing these emotions could be found. Among rare studies are publications by Wierzbicka (2009); among other things she emphasizes that English being de facto the international scientific language may interfere with studying emotions. Prosodial emotions in everyday “unemotional” speech might be least pronounced in English (as a result of the recent 500 years of changes in English grammar and sounds); prosodial emotional functions in English have been taken by songs more than in other languages (Perlovsky, 2009, 2010, 2013). The current state of experimental study of such a fundamental aspect of human psychology as emotionality of everyday speech is inadequate. And in general, measuring aesthetic emotions, their number, properties of their spaces (clusters, evolution with culture), remains elusive.

## Difficulties of measuring aesthetic emotions

Emotions motivate every human action and intention in the world and in the mind (e.g., Markus and Kitayama, 1991). They are among most ancient mental mechanisms. Their cognitive-mathematical models are straightforward (Grossberg and Levine, 1987; Perlovsky, 2001). Still, some scientists perceive emotions as more complex than concepts, emotions sometimes may seem as almost mysterious. This might be related to the fact that emotions are not completely logical and not always completely conscious. Therefore, I start discussing how to measure emotions with standard, usually conscious, everyday emotions.

A classical approach (Shaver et al., 1987) uses emotional words. Shaver first selected near 250 English words with “emotional” content, and had a group of participants to sub-select words designating emotions. This procedure resulted in approximately 140 emotional words. Then he had another group estimating subjective similarity measures between every pair of words. This produced a 140 × 140 matrix of similarity measures, which was used in a procedure similar to multidimensional scaling (Torgerson, 1952). A somewhat different approach was used by Petrov et al. (2012); instead of subjective similarities this approach used objective measures of differences among contexts in which emotional words are used. Results of both studies are similar to many publications identifying relatively few “important emotions” (e.g., see Plutchik, 1962; Scherer, 2005); in Petrov et al. (2012) 5 largest eigenvectors (combinations of emotions) describe about 25% of the “volume” of the emotional space occupied by 130 emotional words (“vectors”). The main point here is that these methods based on emotional words and pair wise similarity-distance measures give a method to analyze objectively properties of emotional spaces, including their dimensionalities (the number of distinct emotions).

The first method in the above paragraph based on subjective similarity can be directly extended to aesthetic emotions. Let me discuss a few difficulties to be expected. Consider first musical emotions. A first hypotheses to test could be that virtually every musical phrase of every significant composer expresses (or creates) a new distinct emotion. The experiment could consist in measuring subjective similarities or differences among a large number of different musical phrases and then establishing dimensionality of the resulting space. The following difficulties can be expected. (1) Differences among musical styles and composers (say Chopin and Eminem) are much stronger and more pronounced than differences, say among various Chopin phrases; it is likely that fine differences among Chopin phrases could be masked by differences among styles and composers. (2) Specifics of fine differences among musical emotions could be fleeting, different among participants, different for the same participant at various times, depending on his/her psychological state, in other words, unrepeatable. But repeatability is a cornerstone of experimental procedures. Some substitute of “usual” repeatability would have to be invented.

The same method could be tried for aesthetic emotions of cognitive dissonances, prosodial emotions, and aesthetic emotions corresponding to visual arts. In addition to discussed difficulties, we can anticipate that (3) emotions reported by participants may be different from those intended to be measured. For example, aesthetic emotions of prosody in usual non-articulated speech might be unconsciously mixed up with much stronger basic emotions in the contents of speech. Similarly, conceptual contents of a visual piece of art could be much stronger than its emotional contents. Or consider cognitive dissonance among two different dishes; differences in imagined gustatory emotions are likely to mask the target emotions of cognitive dissonance (e.g., see Bonniot-Cabanac et al., 2012).

## Approaches to measuring aesthetic emotions

Despite the discussed difficulties I suggest that aesthetic emotions can be measured. Consider again subjective emotional differences among pairs of musical phrases in a large data base. Even as gross emotional differences among styles and composers might mask some aspects of fine differences say between two phrases of Chopin, nevertheless hundreds of thousands of people attentively listen for hours to music of Chopin, or Schubert, or Bach, or Beethoven without losing attention, and music listeners report that the main interest and attraction of music is emotional experience (Zentner et al., 2008). This by itself is a tentative evidence for the hypothesis that every musical phrase brings new emotion.

It might follow that to fine-tune experimental procedure for measuring subjective differences among musical emotions experimental setups could concentrate on fine emotional differences and exclude gross differences, in other words, could explore similar music sets, e.g., a single composer, or even a single piece of music. This will stimulate listeners to concentrate on fine differences, and not get distracted by gross difference in styles, genres, instruments, large orchestra vs. solo, etc. After establishing dimensionalities of emotional spaces of individual musical pieces, the next step could concentrate on an individual composer, and then gradually explore emotional spaces of various composers, styles, genres, etc. In parallel, experimental and mathematical techniques could be developed to explore conjunctions of different emotional spaces.

Aesthetic emotions could differ depending not only on stimuli but also on individual psychological states of a perceiving individual. Therefore, averaging emotional differences over experimental participants may not be appropriate. Possibly measures obtained at different sessions with the same participants might not be averaged either. Diversity of individual perceptions on different occasions might be valid emotional differences. Objective confirmations of the results might be found in similarities of properties of emotional spaces (such as dimensionalities, areas, and volumes of emotional spaces of individual music pieces, composers, etc.). As this kind of experimental data will become available, appropriate measures of statistical significance will be developed.

Alternatively to subjective measures of emotional diversity, experimental procedures might concentrate on comparing musical texts. This approach is somewhat similar to measuring properties of emotional spaces using contexts in Petrov et al. (2012). Using chordal harmonies music notations might be preferable for this purpose.

Another approach to measuring aesthetic emotions can be based on brain imaging (Blood and Zatorre, 2001; Schmidt and Trainor, 2001; Koelsch et al., 2006; Wilkins et al., 2012). Can we identify brain image “signatures” corresponding to different aesthetic emotions? This experimental approach can be helped by recent neural models suggesting mechanisms of aesthetic emotions and brain regions involved (Levine and Perlovsky, 2010; Levine, 2012) as well as by discussions of brain networks involving emotions and cognition (Pessoa, 2014).

## Summary

The wealth of human emotional experience is made by aesthetic emotions. There are possibly thousands of aesthetic emotions. They evolved relatively recently compared to basic emotions designated by specific words. Measuring aesthetic emotions, which may not be designated by specific words, is more complicated than measuring basic emotions. Specific difficulties have been discussed as well as tentative approaches to overcoming these difficulties. I challenge the experimental community to develop procedures for measuring aesthetic emotions beyond words.

### Conflict of interest statement

The author declares that the research was conducted in the absence of any commercial or financial relationships that could be construed as a potential conflict of interest.
